# A Comprehensive Review on Machine Learning in Healthcare Industry: Classification, Restrictions, Opportunities and Challenges

**DOI:** 10.3390/s23094178

**Published:** 2023-04-22

**Authors:** Qi An, Saifur Rahman, Jingwen Zhou, James Jin Kang

**Affiliations:** 1 School of Information Technology, Faculty of Science, Engineering and Built Environment, Deakin University, Geelong, VIC 3216, Australia; 2 Computing and Security, School of Science, Edith Cowan University, Joondalup, WA 6027, Australia

**Keywords:** machine learning, machine learning algorithms, healthcare, mobile health, supervised learning, unsupervised machine learning

## Abstract

Recently, various sophisticated methods, including machine learning and artificial intelligence, have been employed to examine health-related data. Medical professionals are acquiring enhanced diagnostic and treatment abilities by utilizing machine learning applications in the healthcare domain. Medical data have been used by many researchers to detect diseases and identify patterns. In the current literature, there are very few studies that address machine learning algorithms to improve healthcare data accuracy and efficiency. We examined the effectiveness of machine learning algorithms in improving time series healthcare metrics for heart rate data transmission (accuracy and efficiency). In this paper, we reviewed several machine learning algorithms in healthcare applications. After a comprehensive overview and investigation of supervised and unsupervised machine learning algorithms, we also demonstrated time series tasks based on past values (along with reviewing their feasibility for both small and large datasets).

## 1. Introduction

Machine learning is a mechanism that enables machines to learn automatically without explicit programming. The main area of machine learning is to use advanced algorithms and statistical techniques to access the data and predict accuracy instead of a rule-based system. The dataset is a primary component of machine learning accuracy prediction. As a result, the data are more relevant and the prediction is more accurate. Machine learning has been used in different fields, such as finance, retail, and the healthcare industry [1]. The rising use of machine learning in healthcare provides more opportunities for disease diagnosis and treatment [2]. Machine learning has a great feature of continuous improvement for data accurate prediction and classification purposes for disease analysis. The prediction model will learn to make a better decision for accurate prediction as the increasing data are gathered [3]. Patient datasets recorded in electronic healthcare records can be used to enable the extraction of pertinent data using machine learning techniques [4]. Machine learning algorithms can help in disease diagnosis by analysing data and predicting the underlying causes of an illness by employing disease-causing variables from electronic health records [5]. Machine learning gained popularity in terms of classification, prediction, and clustering tasks over the traditional biostatistical approach for analysing and integrating enormous amounts of complicated healthcare data [6]. Machine learning has recently demonstrated outstanding results in a variety of tasks, including the identification of body organs from medical images [7], interstitial lung diseases classification [8], reconstruction of medical images [9,10], and segmentation of brain tumors [10].

Cloud computing, deep learning, artificial intelligence, big data, and machine learning are all used in mobile health (mHealth) nowadays. By using cellular network technologies, wearable sensor devices can transmit health data to hospital databases and then to cloud storage systems. Data collected from these sources can then be analyzed for medical purposes [11]. Many researchers have used machine learning for disease detection and pattern recognition [12]. There have been a number of studies that have examined how multi-layer inference algorithms can improve the trade-off between efficiency and accuracy in data analysis [13]. Despite this, only a limited number of studies have demonstrated that machine learning algorithms can enhance healthcare data accuracy and network efficiency. Thus, this paper aimed to explore and evaluate whether machine learning techniques are practical for enhancing healthcare data metrics like accuracy and efficiency. This study’s primary goal was to fill the knowledge gap in the application of machine learning in healthcare. The following are this paper’s contributions:

- Supervised machine-learning: the papers in this category cover different machine-learning models’ performance and limitations in the healthcare industry.
- Unsupervised machine learning in the healthcare industry: this category covers the advantage and disadvantages of unsupervised models, where labeled data are unavailable
- Comparative analysis of machine learning model: the papers in this category cover all possible machine learning model used in the healthcare industry and their performance which will provide a future direction for the researcher to think more about machine learning-based solutions in healthcare.

## 2. Overview of Machine-Learning in Healthcare

**Figure 1:**
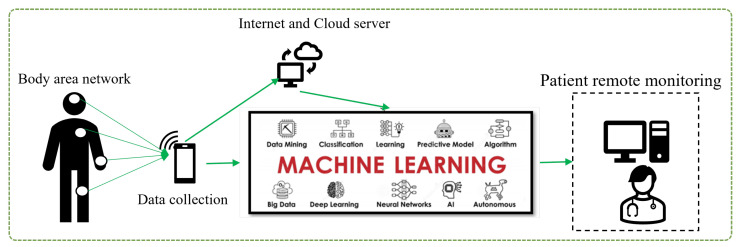
Concept of machine learning in healthcare area.

Machine learning is a type of artificial intelligence that involves training algorithms on data so that they can make predictions or take actions without being explicitly programmed. In healthcare, machine learning has the potential to revolutionize how we diagnose, treat, and prevent diseases, as shown in Figure 1. Some potential applications of machine learning in healthcare include:

- Predictive analytics: Machine learning algorithms can analyze data from electronic health records, claims data and other sources to predict the likelihood of specific health outcomes, such as hospital readmissions or the onset of chronic diseases. This can help healthcare providers identify high-risk patients and take proactive steps to prevent adverse outcomes.
- Diagnosis and treatment: Machine learning algorithms can be trained to analyze medical images, such as CT scans or X-rays, to help diagnose or identify the most appropriate treatment for a patient.
- Personalized medicine: Machine learning can be used to predict which treatments are most likely to be effective for a given patient based on their individual characteristics, such as their genetics and medical history.
- Clinical decision support: Machine learning algorithms can be integrated into clinical decision support systems to help healthcare providers make more informed decisions about patient care.
- Population health management: Machine learning can be used to analyze data from large populations to identify trends and patterns that can inform the development of public health initiatives.

Overall, the use of machine learning in healthcare has the potential to improve patient outcomes, reduce costs, and enhance the efficiency of the healthcare system.

The rest of this paper is organized as below.

## 3. Review of Machine Learning

**Figure 2:**
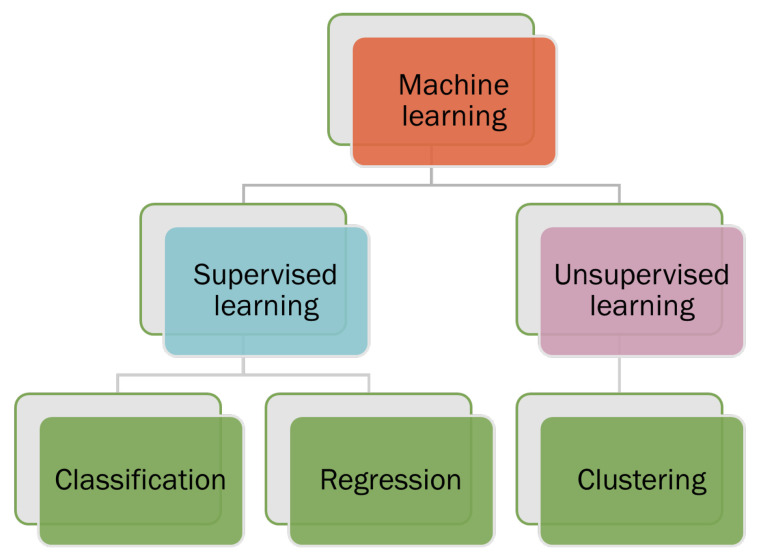
Types of machine learning such as supervised and unsupervised learning.

Machine learning can be categorized into two categories: supervised learning and unsupervised learning, shown in Figure 2. Supervised machine learning trains the algorithms on known input and output data to predict future outputs. Unsupervised machine learning discovers hidden patterns or internal structures within the input data. Supervised machine learning can perform both classifications and regression tasks, while unsupervised machine learning tackles the clustering tasks [14].

### 3.1. Some Common Supervised Classification Machine Learning Algorithms

Supervised machine learning classification techniques are algorithms that predict a categorical outcome called classification, the given data are labelled and known compared to unsupervised learning. The input data are categorised into training, and testing data [15]. The classification algorithms predict discrete responses by classifying the input data into categories. The classical supervised machine learning application includes heart attack prediction, medical image processing, and speech recognition [14]. Supervised learning derives classification models from these training data. These models can then be used to perform classification on other unlabelled data. The training dataset includes an output variable that needs to be classified. All algorithms learn specific patterns from the training data and apply them to the test data for a classification problem [14]. Some well-known supervised classification machine learning algorithms are decision trees, support vector machines, naïve Bayes, K-nearest neighbors, and neural networks.

**Figure 3:**
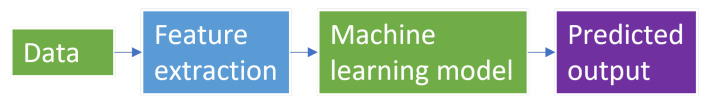
The general architecture of machine learning with requires steps such as data to feature extraction and training to prediction using different machine learning models.

The general machine learning architecture is shown in Figure 3 and the details of this step are described as follows:

#### 3.1.1. Health Datasets

Healthcare datasets are comprehensive collections of information related to patients’ health. These datasets typically contain a broad range of data points, including medical history, diagnostic test results, medication usage, and demographic information. They serve various purposes, such as clinical research, public health monitoring, and quality improvement initiatives. Examples of healthcare datasets include electronic health records (EHRs), which are digital records of patient’s medical information, and claims datasets, which provide information about healthcare services received and their associated costs. Additionally, there are disease registries that contain data on individuals with specific diseases or conditions, and clinical trial datasets that contain information on participants, interventions, and outcomes. Healthcare datasets are complex and can be challenging to analyze due to their size and complexity. Nevertheless, researchers can use advanced analytical techniques such as machine learning and natural language processing to gain insights into patient health outcomes and develop targeted interventions to improve patient care.

#### 3.1.2. Feature Extractions

Selecting the most relevant features from a dataset is a crucial component of machine learning known as feature extraction [16]. Feature extraction involves transforming the raw data into features that possess a strong ability to recognize patterns. In this process, the original data are considered to have weak recognition ability compared to the extracted features [17]. The objective of this process is to identify the vital attributes or traits from the original data that will serve as inputs for a machine learning algorithm to execute a specific task. Numerous methods are available for feature extraction, including principal component analysis (PCA) [18], linear discriminant analysis (LDA) [19], t-distributed stochastic neighbor embedding (t-SNE) [20], autoencoders [21], filter methods [22], and wrapper methods [23].

- PCA: PCA is a widely used dimensionality reduction method in data analysis and machine learning. As a linear transformation approach, it aims to discern patterns in high-dimensional data by projecting it onto a lower-dimensional space. The primary objective of PCA is to encapsulate the most important variations in the data while minimizing noise and redundancy [18].
- LDA: Linear discriminant analysis (LDA) is a supervised dimensionality reduction method extensively used in machine learning, pattern recognition, and statistical evaluation. LDA’s main goal is to convert high-dimensional data into a lower-dimensional space while optimizing the distinction between different classes. This property makes LDA especially fitting for classification tasks, as well as for extracting features and visualizing multifaceted, multi-class data [19].
- t-SNE: t-SNE is a non-linear dimensionality reduction method that is especially adept at visualizing high-dimensional data. Laurens van der Maaten and Geoffrey Hinton created t-SNE in 2008. Its primary purpose is to conserve local structures within the data, which entails preserving the distances between adjacent data points during dimensionality reduction. This characteristic renders t-SNE highly effective in unveiling patterns, clusters, and structures within intricate datasets [20].
- Autoencoders: Autoencoders are a form of unsupervised artificial neural network employed for dimensionality reduction, feature extraction, and representation learning. They comprise an encoder and a decoder that collaboratively compress and reconstructs input data while minimizing information loss. Autoencoders are especially valuable for tasks such as denoising, anomaly detection, and unsupervised pre-training for intricate neural networks [21].
- Filter methods: these techniques prioritize features by evaluating them using specific statistical metrics such as correlation, mutual information, or the chi-square test. Subsequently, the most prominent features are chosen. Filter methods are exemplified by approaches like Pearson’s correlation and Information Gain (IG) method [22].
- Wrapper methods: Wrapper methods represent feature selection approaches utilized in machine learning and data analysis. Their main objective is to determine the best subset of features that enhances the performance of a specific machine learning algorithm. By directly evaluating various feature combinations based on the performance of the learning algorithm, wrapper methods are more computationally demanding than filter methods, which are based on the data’s inherent characteristics [23].

#### 3.1.3. Decision Trees

**Figure 4:**
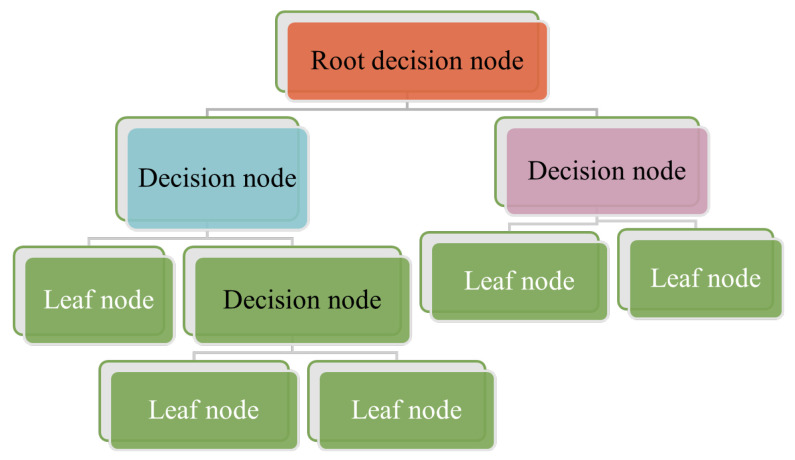
Demonstration of decision trees with the root, decision, and Leaf nodes. Start from the root node, then move to the decision node using the leaf node information.

A decision trees classifier uses graphical tree information to demonstrate possible alternatives, outcomes, and end values (Figure 4). This involves a computational process to calculate probabilities in deciding on a few courses of action [24]. The decision trees algorithm starts with training data samples and their related category labels. The training set is recursively divided into subsets based on feature values, so the data in each subset is purer than the data in the parent set. Each internal node of the decision tree represents a test feature, whilst every branch node presents the test result, and the leaf nodes present the class label. Since the classifier decision tree is used to identify an unknown sample’s category label, it will be able to track the path from the root node to the leaf nodes and hold the sample’s category label [25]. The advantage of the decision tree algorithm is that it is fast and simple, where no domain knowledge or parameter setting is required, and high dimensional data can be handled in the context. Further, decision tree algorithms support incremental learning, which is immutable because of the alternative functions based on each internal node [25].

Building decision trees can be a lengthy process, particularly when working with sizable datasets or a high number of features. This is because the algorithm must evaluate every potential split at each level of the tree, which can be computationally costly [26].

Medical experts frequently employ data mining techniques to aid in the diagnosis of cardiac disorders. Regarding sensitivity, specificity, and accuracy, the decision tree is one of the effective machine-learning algorithms for heart attack detection [27]. In the medical field, heart disease has been extensively detected and prevented using the decision tree classification technique. Using eight patient data variables and a decision tree, Pathak and Valan were able to predict heart disease with an accuracy of 88% [28], while [29] used the decision tree for prediction of heart disease. In [24], researchers employed decision tree algorithms to reduce the volume of data by converting data into a more condensed form in order to preserve the crucial features and increase accuracy in mobile health technology.

#### 3.1.4. Support Vector Machine (SVM)

**Figure 5:**
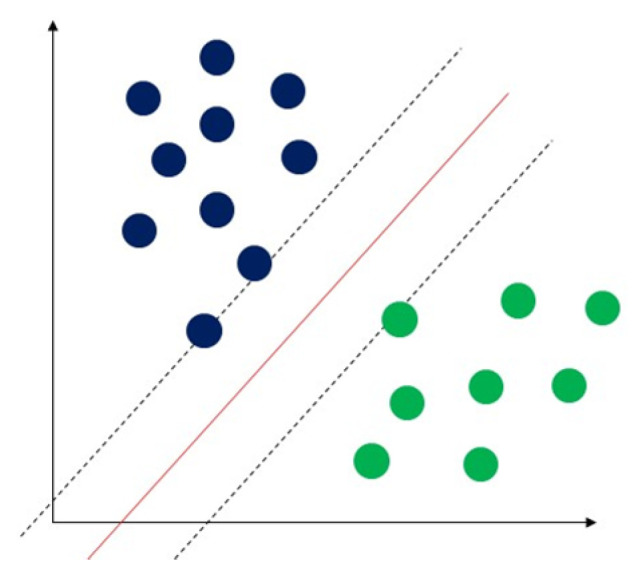
Demonstration of support vector machine. The solid red line indicates the separating hyperplane and the distance between two dotted lines is the maximum margin for separating different classes.

Support vector machine (SVM) is a classical machine learning technique that can address classification problems. Importantly, SVM supports multidomain applications in a big data mining environment [30]. SVM uses some model features to train data to generate reliable estimators from a dataset [31]. The concept of SVM maximizes the minimum distance from the hyperplane to the nearest point of the sample presented in Figure 5 [32].

SVM produces higher performance when dealing with a large dataset than other pattern recognition algorithms, such as Bayesian networks, etc. Additionally, one main advantage of SVM is that its data training is comparatively easy (Pradhan, 2012). Most importantly, according to (Bhavsar and Ganatra, 2012), SVM provides high accuracy among machine learning algorithms. The disadvantage of SVM is that it is exceedingly slow in machine learning, as a large amount of training time is needed. Further, memory requirements increase with the square of the number of training examples [33]. SVM is one of the most effective machine learning algorithms for pattern recognition. Most SVM applications are used for facial recognition, illness detection and prevention, speech recognition, image recognition, and facial detection [34]. Some authors have used an improved stacked SVM for early heart failure (HF) prediction in medical applications. Their findings demonstrated that the model has superior performance with an accuracy range from 57.85% and 91.83% [35]. In a different study, fuzzy support vector machines were utilized to make diagnoses of coronary heart disease. Experiments revealed that, when compared to non-incremental learning technology, this technique significantly sped up illness diagnosis computation time [36].

#### 3.1.5. Naïve Bayes

**Figure 6:**
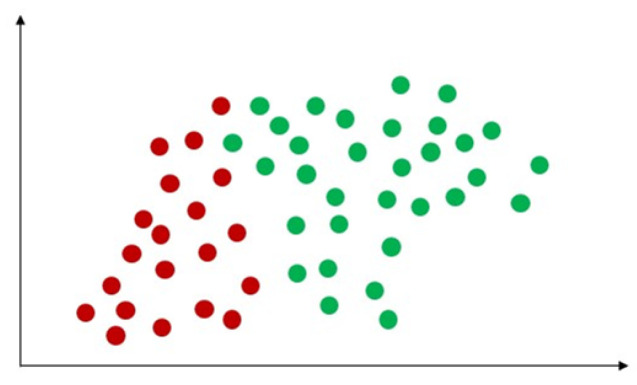
Demonstration of naïve Bayes with the distribution of different classes.

Naïve Bayes is one of the most widely used classification algorithms. The assumption of naïve Bayes only includes one parent node and a few independent child nodes rendering it the simplest Bayesian network [37]. Naive Bayes (NB) uses the probability classification method by multiplying the individual probability of each attribute-value pair, as shown in Figure 6. This simple algorithm presumes independence between attributes and provides remarkable classification results [38]. One strength of the naïve Bayes algorithm is that it has a short computational data training time [39], where classification performance can be improved by removing irrelevant attributes [40]. This can lead it to perform better with small datasets and in dealing with multiple classification tasks. In addition to this, naïve Bayes is suitable for incremental training (where it can train supplementary samples in real-time) [41]. As the algorithm is not very sensitive to missing data, is relatively simple, and can often be used for text classification, naïve Bayes is easy to understand the interpretation of the results [42]. The drawbacks of the naïve Bayes include its lower rate of accuracy compared to other sophisticated supervised machine learning algorithms, such as ANNs. Further, naïve Bayes requires many training records to achieve excellent performance results [43]. Since naïve Bayes is very efficient and easy to implement, it is commonly used in text classification, spam filtering, or news classification [44]. In the medical field, the naïve Bayes algorithm has been used for disease detection and prediction. One study deployed a naïve Bayes classifier to skin image data for skin disease detection, revealing the results to outperform other methods with accuracy from 91.2 to 94.3% [45]. Gupta et al. have used naïve Bayes for heart disease detection through feature selection in the medical sector, with experimental results achieving 88.16% accuracy in the test dataset [46].

#### 3.1.6. K-Nearest Neighbours (K-NN)

**Figure 7:**
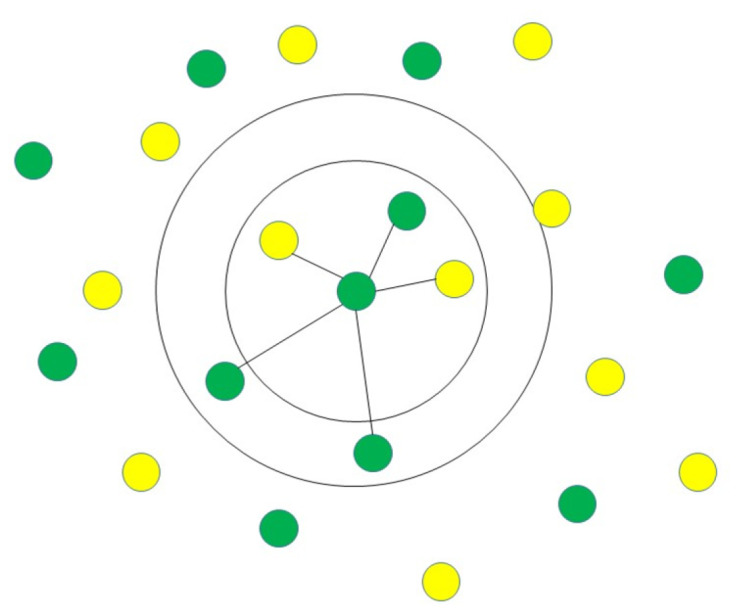
Demonstration of K-NN identifying unknown pattern by assigning a value to the K, where the nearest neighbor category of the K training sample is considered the same as the classification.

The K-nearest neighbours (K-NN) classification algorithm is one of the simplest methods in data mining classification technology. The assumption of K-NN is to identify an unknown pattern by assigning a value to the K, where the nearest neighbor category of the K training sample is considered the same as the classification illustrated in Figure 7 [47]. A few factors are involved in the classifier, such as selected K-value and distance measurement, and so on [48]. K-NN requires less computational time to train the data than other machine algorithms. However, it requires more computational time in the classification phase [33]. The advantage of K-NN is that it is easy to understand and implement for classification. Further to this, it can perform well with many class labels for a dataset. Similarly, the data training stage is faster than other machine learning algorithms [33]. The drawbacks of the K-NN are its computational cost, with a sizeable unlabelled sample, and time delay during the classification phase. Apart from cross-validation, k-NN also lacks the principles to sign a K’s value and is expensive computationally. Further, confusion may occur if too many unrelated attributes are in the data, leading to poor accuracy [33]. K-NN is also frequently utilized for disease detection and diagnosis [49]. K-NN is one of the most used data mining approaches for classification problems, and researchers have tried to utilize it to help medical professionals diagnose heart disease [50]. To identify heart disease, for instance, some researchers have developed a unique algorithm that combines K-NN and genetic algorithms in an effort to increase the accuracy of prediction [49]. Shouman et al. studied whether incorporating other algorithms into K-NNs can improve accuracy in the diagnosis of cardiac disease. According to their findings, using K-NN instead of a neural network could increase the accuracy of diagnosis of heart disease [50]. The summary of existing supervised learning performance in terms of accuracy in the healthcare industry using classification algorithms is presented in Table 1.

## 4. Some Popular Supervised Machine Learning Regression Algorithms

Supervised regression techniques are algorithms that can predict a continuous response, known as regression techniques [51]. The goal of the supervised regression task is to forecast an outcome’s specific value rather than to classify the data [13]. Input data are split into training and testing data, where the continuous response or target outcome is predicted by selected algorithms [15]. Typical regression techniques are used in algorithmic trading and electricity load forecasting [51]. The most popular regression machine learning algorithms are linear regression, logistic regression, ensemble methods, and support vector regression (SVR), as discussed below.

### 4.1. Linear Regression

**Figure 8:**
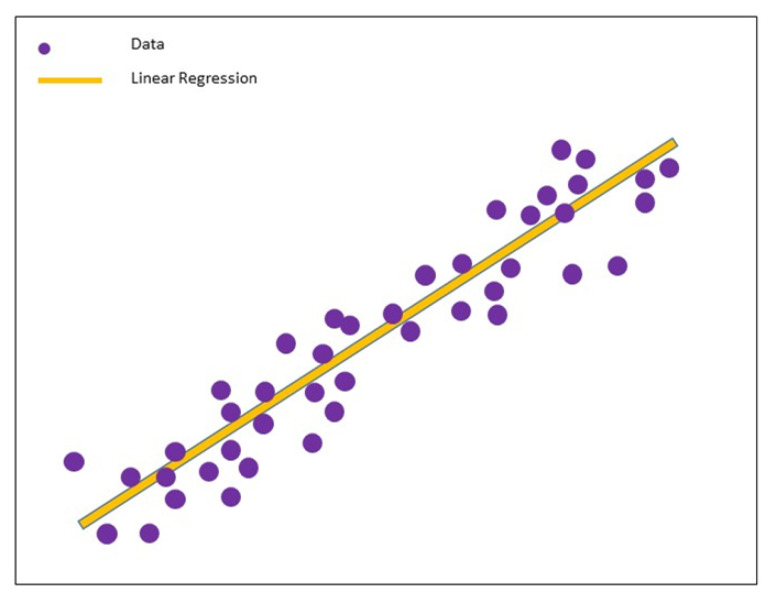
Demonstration of linear regression with best-fit line.

The linear regression technique is the most simple and desired method to measure the relationship between response variables and continuous predictors. Linear regression assumes that the predictor and target variables have a linear relationship, as shown in Figure 8. Its simplicity makes the linear regression technique the best option for small sample analysis with high accuracy, where it is comparatively easy to understand and interpret. However, this model may not achieve the expected result if there are too many predator variables [52]. Further, as it involves a one-to-one relationship between variables, it is not a good fit when dealing with non-linear relationship data [26], where most problems involve non-linear characteristics to differing extents. Linear regression is also unsuitable for highly non-linear problems when the relationship cannot be approximated by a linear function between input and output variables. However, before applying other complex machine learning algorithms, it may be worthwhile to try linear regression or other simple machine learning algorithms to understand the difficulty of a problem [53]. Authors in [54] use linear regression to achieve healthcare resource utilization.

### 4.2. Logistic Regression

**Figure 9:**
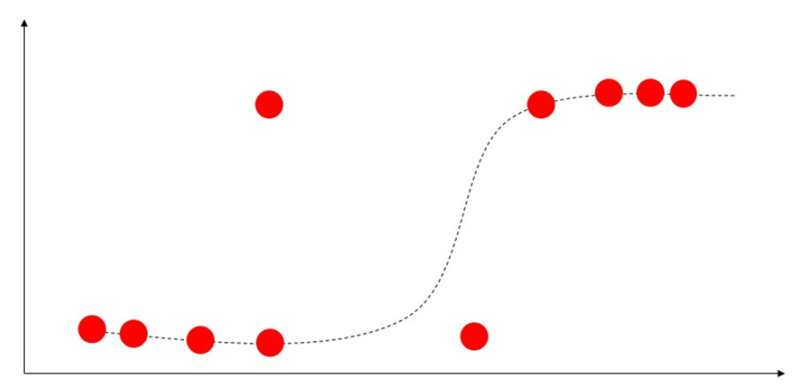
Demonstration of logistic regression with s-curve line.

Unlike linear regression, which is used to predict continuous quantities, logistic regression is mainly used to predict discrete class labels. A logistic regression algorithm predicts probability with two possible categories for classification problems. Logistic regression uses a logistic function to classify the label in a binary outcome between 0 and 1 presented in Figure 9. Therefore, the output variable can be used to indicate which category a sample belongs to [53]. The authorsofin [55] use logistic regression to predict health-related behaviours.

### 4.3. Ensemble Methods

**Figure 10:**
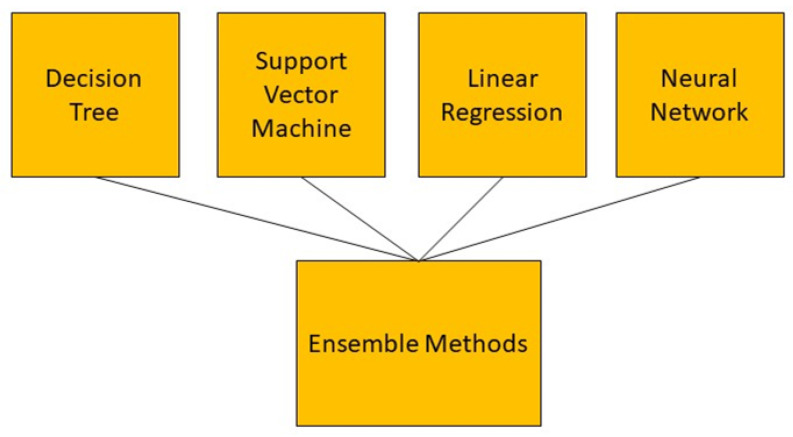
Demonstration of ensemble methods which combine the different machine learning algorithms.

Ensemble methods are not a single machine learning algorithm, rather, they combine the strength of other algorithms. This can complete learning by constructing and combining multiple machine-learning devices (Figure 10). One advantage of ensemble methods is that they have high predictive accuracy that can be achieved in machine learning; however, the model’s training process may be more complicated, where efficiency may not be possible. There are two standard ensemble learning algorithms currently: bagging-based algorithms and boosting-based algorithms. Bagging-based representative algorithms include random forest and boosting-based representative algorithms include Adaboost, GBDT, and XGBOOST [56]. There are a few advantages to using ensemble methods. Firstly, they can avoid the overfitting problem. A single machine learning algorithm can easily find many different hypotheses that can ideally forecast all the training data with less accuracy prediction for unseen examples when using a small data size. Thus, using combined algorithms (the different hypotheses of Averaging) minimizes the risk of selecting unsuitable hypotheses, thus improving overall forecasting performance. Secondly, ensemble methods have the advantage of computation. Ensemble methods can reduce the risk of reaching a local minimum by combining several algorithms as a single algorithm to perform a local search that may fall into the optimal solution. In any single model of an algorithm, the optimal hypothesis may go outside of space. Ensemble methods can extend the search space to fit the data by integrating different algorithm models. The ensemble method can suit complex problems with large datasets [57]. The ensemble methods were used in [58] to predict patients’ weekly average expenditures on certain pain medications.

### 4.4. Support Vector Regression (SVR)

**Figure 11:**
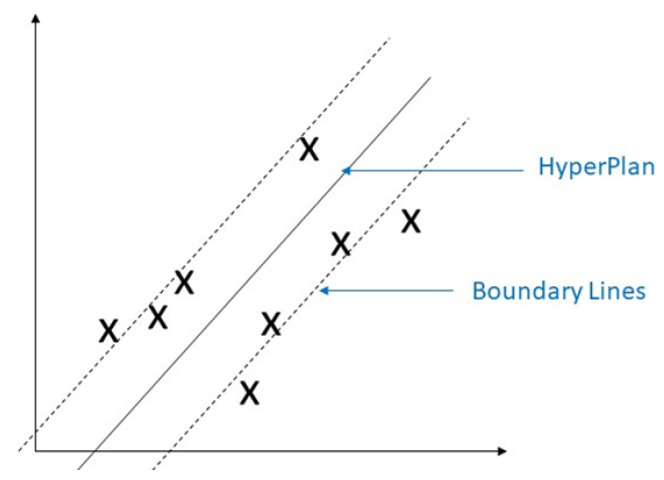
Demonstration of support vector regression. The solid black line indicates the separating hyperplane, and the distance between two dotted lines is the boundary line for separating different classes.

Support vector regression (SVR) is a supervised regression technique used to study the relationship between one or more independent variables and a dependent variable (continuous value) shown in Figure 11. Unlike linear regression techniques that rely on model assumptions, SVR learns the importance of variables to characterize the relationship between input and output [59]. SVR attempts to seek a linear regression function that can maximally approximate the vector of actual data output with tolerance of error [60]. One of the primary advantages of SVR is that its complexity of computation does not rely on the dimensionality of the input variables. In addition, it has incredible generalization ability and high prediction accuracy [61]. However, SVM is expensive computationally and requires a large dataset. In study [62], the authors used SVR to visualize and predict the COVID-19 outbreak. The summary of existing supervised learning performance in terms of accuracy in the healthcare industry using regression algorithms is shown in Table 2.

## 5. Unsupervised Machine Learning

Unsupervised machine learning techniques are used to analyze large amounts of unlabelled data with highly non-linear learning, using millions of parameters of complex models [63]. As a common clustering learning technique, this technique can be used to group or find hidden patterns in data for exploratory data analysis. Unsupervised machine learning draws inferences from datasets, including input data without labelled responses. Most unsupervised learning applications are used for market research, gene sequence analysis, and object recognition [64]. One of the fundamental rules of unsupervised learning is grouping data into suitable groups. While clustering analysis is used with the same attributes, the formal approach and techniques used to cluster and categorise the data are based on similarities and properties of the objects. This does not entail categorising labels with categories, which is how data clustering differs from it in the absence of category information [63]. There are two categories of clustering algorithms: soft clustering and hard clustering. Hard clustering occurs when data points belong to one cluster, whereas data points belonging to one or more clusters are referred to as soft clusters. Some popular unsupervised machine learning algorithms are discussed below.

### 5.1. Common Hard Clustering Algorithms

#### 5.1.1. K-Means

**Figure 12:**
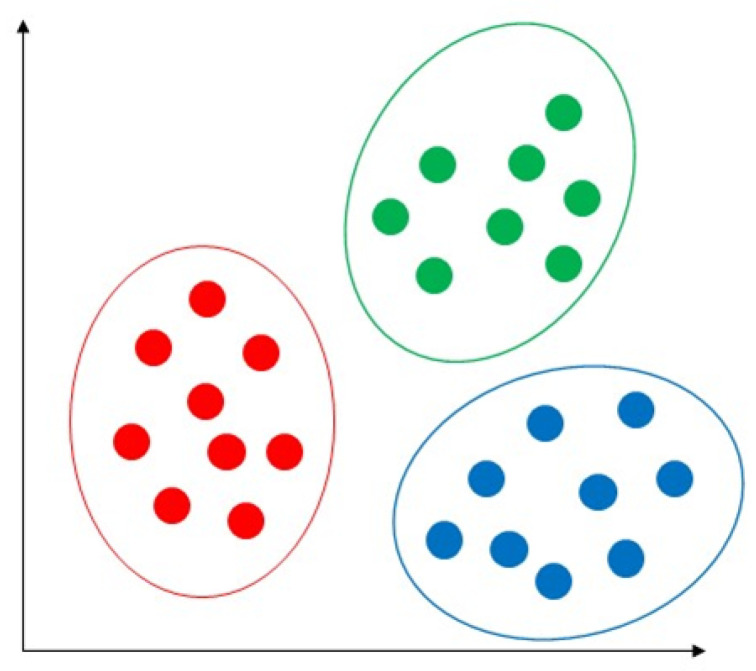
Demonstration of K-means algorithm by the partition of data points into k clusters by minimizing the sum of the squared distance between the point.

K-means is an extensively used unsupervised algorithm [63] where its simplicity and fast speed [65] allow it to solve well-known clustering problems [66]. The K-means algorithm partitions data points into k clusters by minimizing the sum of the squared distance between the point [67,68] and its nearest neighbor set distance as shown in Figure 12. The matching degree between a point and the cluster depends on the distance from the point to the cluster center [69]. The best use of the K-means algorithm is when the number of clusters is known for fast clustering with a large number of datasets [66]. Therefore, K-means remains the most well-known population for massive datasets analysis in unsupervised learning [69]. In practice, the advantages of the K-means algorithms include: being easy to learn, fast training speed, and no requirement to follow the input data order, and its “vector quantization” concept can be used to construct a feature [68]. This algorithm can adjust the cluster membership for unsupervised clustering learning tasks [70]. K-means has the disadvantages of sensitivity to outliers and scale of datasets; requirements for specifying the number of clusters in advance, resulting in different outcomes with different initial centroids; and the inability to handle density and varying size of convex clusters [70]. The authors of [71] used K-means to predict heart disease and achieved 88% accuracy.

#### 5.1.2. K-Medoids

**Figure 13:**
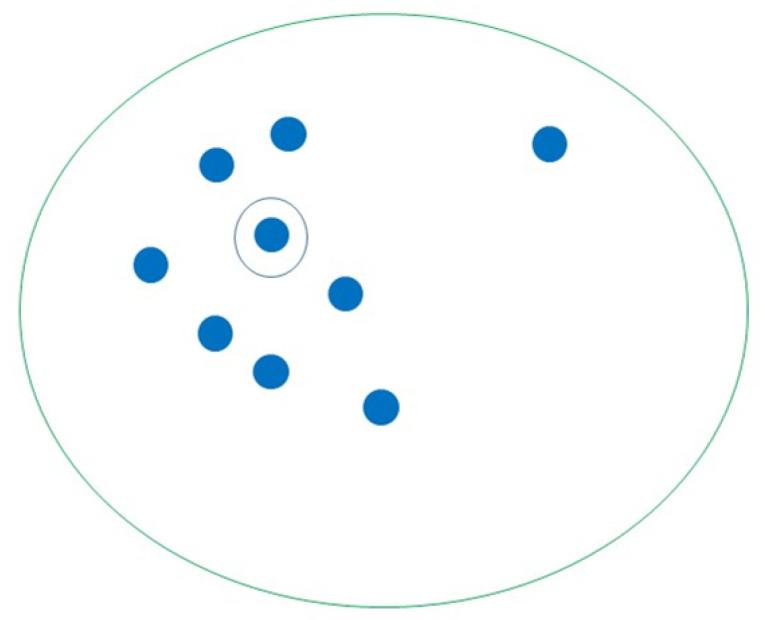
Demonstration of K-medoids through finding the most central object within the cluster and assigning the nearest object to the medoids to create a cluster as a reference point.

K-medoids is similar to K-Means but uses an actual object to find the most central object within the cluster and assign the nearest object to the medoids to create a cluster instead of using the mean value of an object in the cluster as a reference point (Figure 13). K-medoids is less sensitive to outliners and can adjust cluster membership, and it has a similar limitation of producing different results with different initial centroids. Further, it employs best practices when scaling to large datasets, fast clustering of categorical data, and the number of clusterings is known [70]. The researchers in [72] used K-medoids to detect anomalies in smart healthcare.

#### 5.1.3. Hierarchical Clustering

**Figure 14:**
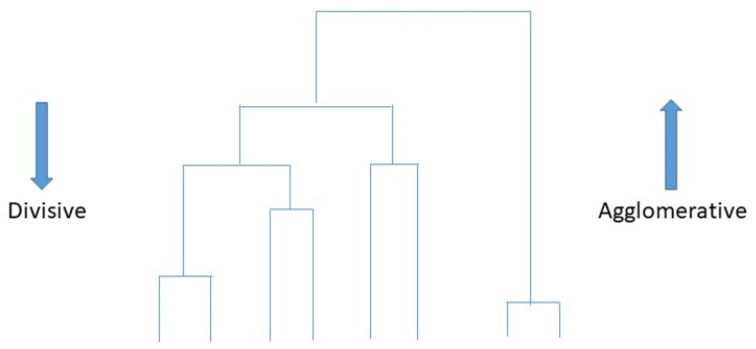
Demonstration of hierarchical clustering by analysing similarities of the characteristics in clusters.

Hierarchical cluster analysis (HCA), also called hierarchical clustering (Figure 14), is a typical cluster analysis method in data mining, which attempts to establish a hierarchical structure of clusters by analysing similarities of the characteristics in clusters [73]. The hierarchical clustering technique recursively produces nested sets of clusters in a dendrogram with cluster [70]. Two strategies are used in hierarchical cluster analysis: agglomerative and divisive strategy. The agglomerative clustering strategy approach is known as “bottom to up”, directing “the leaves” to “the root” of a cluster tree. Contrastingly, the divisive clustering approach is “top-down”, directing “the root” to “the leaves”. All observations are initially treated as one cluster, and then splits occur when moving down into the hierarchical structure [73]. Hierarchical clustering can detect different sizes and shapes within datasets. There is no requirement to specify the number of clusters in advance, forming a dendrogram graphical visualisation when it is not sure how many clusters are in the data. Conversely, this approach has the disadvantages of high complexity and low speed due to expensive computation, where no adjustments can be made after the clustering task. In addition to this, it is not easy to decide the dendrogram level in this approach, where clusters reply on the distance metric used [70]. Hierarchical clustering is used in [74] for mental health prediction and achieves 90% accuracy.

### 5.2. Some Common Soft Clustering Algorithms

#### 5.2.1. Fuzzy c-Means

**Figure 15:**
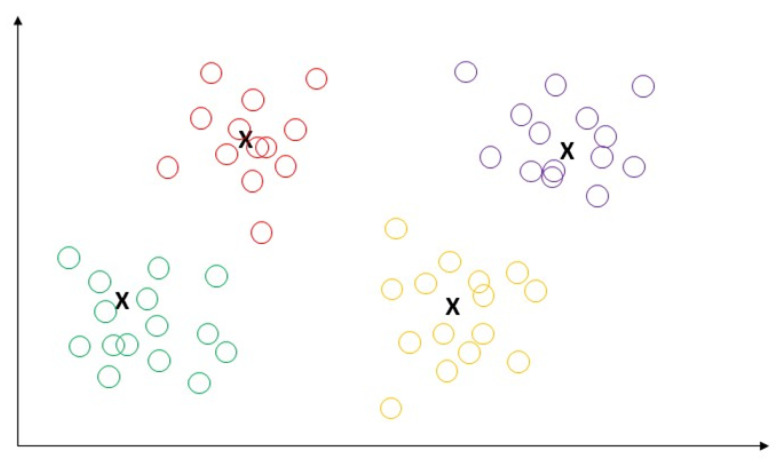
Demonstration of fuzzy c-means by grouping the data into N clusters when clusters overlap.

The fuzzy c-means clustering algorithm is a popular approach that clusters data points when it belongs to more than one cluster. This is similar to the K-means but is suitable for pattern recognition when clusters overlap. The strength of fuzzy c-means is in how it allows clusters to assign flexibility, where it is more practical to provide the probability of belonging to a cluster, as shown in Figure 15. However, this algorithm has some weaknesses relating to high complexity in specifying the number of clusters in advance [70]. Authors in [75] use fuzzy c-means to analyze patient satisfaction perception and achieve 76% accuracy.

#### 5.2.2. Gaussian Mixture Model

**Figure 16:**
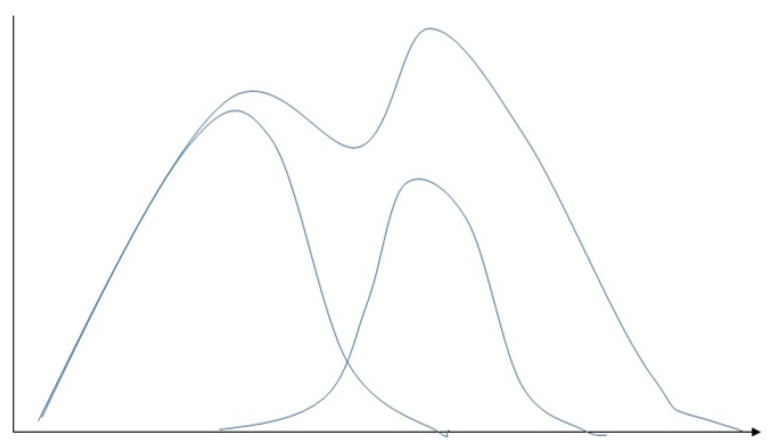
Demonstration of Gaussian mixture model involves representing the probability density function as a blend of several Gaussian distributions, with each distribution corresponding to a cluster present in the data.

The Gaussian mixture model (GMM) is an extension of a single Gaussian probability density function (Figure 16) [76], which uses multiple Gaussian probability density functions (normal distribution curves) to quantify the distribution of variables accurately. This decomposes the variable distribution into several Gaussian probabilities of the statistical model of densities function (normal distribution curve) distribution [77]. The Gaussian mixture model assigns a few single Gaussian distributions, where each of the Gaussian distributions is known as a component with its evaluation index—covariance and mean. The model adjusts the means, coefficients, and covariance through a sufficient number of Gaussian distributions to approximate any continuous function of density closely [78]. The Gaussian mixture model can effectively capture the internal correlation structures within datasets [79]. When data points come from different multivariate normal distributions with specific probabilities and belong to more than one cluster, clustering based on Gaussian mixture is partition-based [76]. The Gaussian mixture model is a flexible model for a wide range of distribution probabilities [64] The feature of clusters can be a few parameters [70]. In addition, it has high accuracy and real-time implementation [80]. The drawbacks of the Gaussian mixture model relate to it being computationally expensive with large distributions or with few observed data points in datasets. Further, it can be difficult to estimate the number of clusters, which requires large datasets [79]. The gaussian mixture model is used in [81] for anomaly detection.

#### 5.2.3. Hidden Markov Model

**Figure 17:**
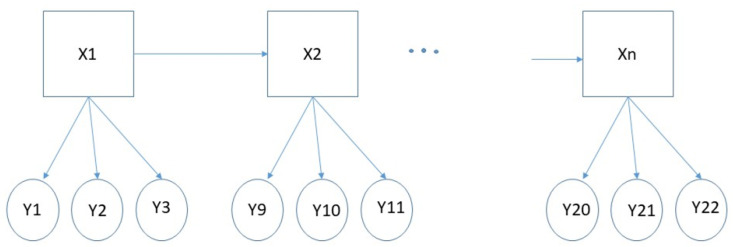
Demonstration of hidden Markov model for a sequence of hidden states over time.

The hidden Markov model belongs to the clustering model-based, valuable, and suitable for time series data. Each data point represents the observer value according to the time sequence by using the hidden Markov model. Future values are clustered based on a time series past value (observed value). The hidden Markov model includes two sections. The first section is the time series observation that generates the observation, followed by the second section-unobserved state variables [70]. A set of states features the model, and the state-related probability distribution manages to generate time-series data. The related state is the first stage—the initial probability distribution, the transition probability matrix connecting successive states, and the dependent probability distribution state (Figure 17). Observers can only see time-series observations, while state variables are hidden. The hidden Markov model provides statistical information such as the standard deviation, mean, and weight value of a cluster based on the cluster’s observation results. The hidden Markov model can deal with a variety of types of data. However, this algorithm requires many parameters and is mostly/only suitable for large datasets [70]. The hidden Markov model was used in [82] to achieve a 70% accurate healthcare audio event classification.

The summary of existing unsupervised learning performance in terms of accuracy in the healthcare industry is presented in Table 3.

## 6. Evaluation of Matrix for Machine Learning

### 6.1. Evaluation Matrix of Supervised Classification Algorithms

The performance of supervised classification algorithms is commonly evaluated by accuracy, sensitivity, and specificity. Accuracy assesses the percentage of prediction rate in the model; sensitivity is the amount of the true positive data points are identified correctly in actual positive data points, and specificity is the quantity of the true negative data points identified in actual negative data points [83] (TP = true positive, TN = true negative, FN = false negative, FP = false positive).

(1) $$Accuracy=\frac{TP+TN}{TP+FN+TN+FP};$$

(2) $$sensitivity=\frac{TP}{TP+FN};$$

(3) $$Specificity=\frac{TN}{TN+FP}.$$

### 6.2. Evaluation Matrix of Supervised Regression Algorithms

For the regression tasks, Mean Absolute Error (MAE), MSE (Mean Squared Error), and Root Mean Squared Error (RMSE) are commonly used to measure the model performance.

**MSE (Mean Squared Error)** is a commonly used metric for comparing predicted values to their corresponding actual values. MSE can be defined by

(4) $$MSE=\frac{1}{n}\sum_{i=1}^{n}{\left(y_{i}-{\hat{y}}_{i}\right)}^{2},$$

where *n* is the number of data points, $y_{i}$ is the actual value for the *i*th data point, and ${\hat{y}}_{i}$ is the predicted value for the *i*th data point.

**RMSE (Root Mean Squared Error)** measures the difference between a set of predicted values and the corresponding actual values, which is similar to MSE but takes the square root of the average of the squared differences. The RMSE is expressed as:

(5) $$RMSE=\sqrt{\frac{1}{n}\sum_{i=1}^{n}{\left(y_{i}-{\hat{y}}_{i}\right)}^{2}},$$

where *n* is the number of data points, $y_{i}$ is the actual value for the *i*th data point, and ${\hat{y}}_{i}$ is the predicted value for the *i*th data point.

**MAE (Mean Absolute Error)** is a measure of the difference between a set of predicted values and the corresponding actual values, which is calculated as the average of the absolute differences between the predicted values and the actual values. The MAE is expressed as:

(6) $$MAE=\frac{1}{n}\sum_{i=1}^{n}\left|\left(y_{i}-{\hat{y}}_{i}\right)\right|,$$

where *n* is the number of data points, $y_{i}$ is the actual value for the *i*th data point, and ${\hat{y}}_{i}$ is the predicted value for the *i*th data point.

**MAPE (Mean Absolute Percentage Error)** is a measure of the accuracy of a prediction or a model, which is calculated as the average of the absolute percentage differences between the predicted values and the actual values. The MAPE is expressed as:

(7) $$MAPE=\frac{1}{n}\sum_{i=1}^{n}\left|\left(y_{i}-{\hat{y}}_{i}\right)/y_{i}\right|\times 100,$$

where *n* is the number of data points, $y_{i}$ is the actual value for the *i*th data point, and ${\hat{y}}_{i}$ is the predicted value for the *i*th data point. These are commonly used performance evaluation metrics in various fields, particularly in forecasting, time series, and medical data analysis using regression algorithms.

### 6.3. Evaluation Matrix of Unsupervised Clustering Algorithms

Evaluating the performance of clustering algorithms is crucial as it is part of data analysis. The evaluation matrix has developed well in supervised learning and is widely accepted. Unlike supervised learning, its evaluation matrix has not developed well, so it is not easy to define the performance of algorithms. However, some indicators can be used to assess the quality of the model. SSE (sum of squared errors) can be used to calculate the Euclidean distance. The smaller SSE value means a good cluster performance. The Calinski–Harabaz index is also called the variance ratio criterion, a metric based on dispersion within clusters and between clusters. The silhouette coefficient is used to define the interval with −1 and 1. Rand index and Fowlkes–Mallows scores (FMI) are used for external criteria validation [84].

## 7. Discussion

Supervised and unsupervised are machine learning methods that have shown great potential in healthcare. Each type has its strengths and limitations, and their applications in healthcare vary based on the type of data and task at hand.

Supervised learning involves training a model with labeled data, where the model learns to predict the outcome based on the input features [85]. In healthcare, supervised learning has been widely used for classification, diagnosis, and prognosis prediction tasks [86]. For example, supervised learning algorithms such as decision trees, support vector machines, and logistic regression have been used to predict the risk of cardiovascular disease, identify cancerous cells, and classify medical images [87]. However, supervised learning requires a large amount of labeled data and may suffer from bias if the training data does not represent the population [88].

On the other hand, unsupervised learning involves training a model with unlabeled data, where the model learns to identify patterns and relationships in the data without explicit guidance [89]. Unsupervised learning has been used in healthcare for clustering, anomaly detection, and feature extraction tasks [90]. For example, unsupervised learning algorithms such as K-means clustering have been used to group patients with similar characteristics, identify rare diseases and extract relevant features from medical images [71]. However, unsupervised learning may be difficult to interpret, and the results may not always be clinically meaningful.

To summarize, supervised and unsupervised learning both have unique strengths and limitations in healthcare. The choice of which type of learning to use depends on the specific task, available data, and resources. As healthcare data grow, machine learning will be essential in improving patient outcomes and advancing medical research.

## 8. Conclusions and Future Work

Healthcare could undergo a variety of technological revolutions because of machine learning. It can increase the precision of the diagnosis, assist in finding patterns and trends in patient data, simplify administrative procedures, and enable individualized treatment regimens. However, there are difficulties with applying machine learning in healthcare, such as issues with data privacy, ethical issues, and the requirement for rigorous validation and regulation. Overall, a deep understanding of the intricate and constantly evolving healthcare landscape, collaboration between healthcare professionals and data scientists, and a dedication to using machine learning ethically and responsibly for the benefit of patients are necessary for successfully integrating machine learning in healthcare.

## Figures and Tables

**Table 1:** Summary of existing supervised learning performance in terms of accuracy in the healthcare industry using classification algorithms.

Classification Algorithms	Reference	Year	Task	Accuracy
Decision trees	[28]	2020	Heart disease prediction	88%
	[24]	2012	Data volume reduction	80/32%
	[29]	2001	Hear disease prediction	81.11%
Support vector machine (SVM)	[35]	2019	Facial recognition, illness detection and prevention, speech recognition, image recognition, and facial detection	57.85–91.3%
Naïve Bayes	[45]	2020	Skin disease detection	91.2–94.3%
	[46]	2020	Heart disease detection	88.16%
	[29]	2001	Hear disease prediction	81.48%
K-nearest neighbours (K-NN)	[49]	2013	Heart disease diagnosis	75.8–100%
	[50]	2012	Heart disease diagnosing	94–97.1%

**Table 2:** Summary of existing supervised learning performance in terms of accuracy in the healthcare industry using regression algorithms.

Regression Algorithms	Reference	Year	Task	Accuracy
Linear regression	[54]	2019	Healthcare resource utilization	95%
Logistic regression	[55]	2003	Predict health-related behavior	87.7%
Ensemble methods	[58]	2020	Predict patients’ weekly average expenditures on certain pain medications	78–98%
Support vector regression (SVR)	[62]	2022	Visualizing and predicting the COVID-19 outbreak	94%

**Table 3:** Summary of existing unsupervised learning performance in terms of accuracy in the healthcare industry.

Common Hard Clustering Algorithms	Reference	Year	Task	Accuracy
K-means	[71]	2021	Heart disease prediction	88%
K-medoids	[72]	2021	Anomaly detection in smart healthcare	75.89%
Hierarchical clustering	[74]	2018	Mental health prediction	90%
**Some Common Soft Clustering Algorithms**	**Reference**	**Year**	**Task**	**Accuracy**
Fuzzy c-means	[75]	2019	Analysis of patient satisfaction perception	76%
Gaussian Mixture Model	[81]	2021	Anomaly Detection	95.5%
Hidden Markov Model	[82]	2020	Healthcare audio event classification	70%

## Data Availability

Not applicable.
